# A systematic review of magnetic resonance imaging in patients with an implanted vagus nerve stimulation system

**DOI:** 10.1007/s00234-021-02705-y

**Published:** 2021-04-12

**Authors:** S. Fetzer, M. Dibué, A. M. Nagel, R. Trollmann

**Affiliations:** 1 Medical Affairs Europe, Neuromodulation, LivaNova Deutschland GmbH, LivaNova PLC owned subsidiary, Lindberghstraße 25, 80939 Munich, Germany; 2 Department of Neurosurgery, Heinrich Heine University, Düsseldorf, Germany; 3 Division of Experimental Radiology, Department of Radiology, Friedrich-Alexander Universität Erlangen-Nürnberg, Erlangen, Germany; 4 Division of Pediatric Neurology, Department of Pediatrics, Friedrich-Alexander Universität Erlangen-Nürnberg, Loschgestr. 15, 91054 Erlangen, Germany

**Keywords:** Depression, Epilepsy, MRI, Vagus nerve stimulation, VNS, Side effects

## Abstract

**Purpose:**

Vagus nerve stimulation (VNS) is an effective adjunctive treatment for drug-resistant epilepsy (DRE) and difficult-to-treat depression (DTD). More than 125.000 patients have been implanted with VNS Therapy® System (LivaNova PLC) since initial approval. Patients with DRE often require magnetic resonance imaging (MRI) of the brain during the course of their disease. VNS Therapy System devices are labeled to allow MRI under certain conditions; however, there are no published comprehensive articles about the real-world experience using MRI in patients with implanted VNS devices.

**Methods:**

A systematic review in accordance with PRISMA statement was performed using PubMed database. Full-length articles reporting MRI (1.5 T or 3 T scanner) of patients with implanted VNS for DRE or DTD and published since 2000 were included. The primary endpoint was a positive outcome that was defined as a technically uneventful MRI scan performed in accordance with the VNS Therapy System manufacturer guidelines and completed according to the researchers’ planned scanning protocol without harm to the patient.

**Results:**

Twenty-six articles were eligible with 25 articles referring to the VNS Therapy System, and 216 patients were included in the analysis. No serious adverse events or serious device-related adverse events were reported. MRI scan was prematurely terminated in one patient due to a panic attack.

**Conclusion:**

This systematic review indicates that cranial MRI of patients with an implanted VNS Therapy System can be completed satisfactorily and is tolerable and safe using 1.5 T and 3 T MRI scanners when performed in adherence to the VNS manufacturer’s guidelines.

**Supplementary Information:**

The online version contains supplementary material available at 10.1007/s00234-021-02705-y.

## Introduction

Epilepsy and depression are prevalent neurological and psychiatric diseases that are often associated with a pharmaco-refractory course, high long-term morbidity, and decreased quality of life [1].

In children and adults, epilepsy is associated with a drug-resistant course in more than 30% of the patients [2]. Patients with DRE [3] are at risk of physical and psychological comorbidities as well as psychosocial problems in addition to their seizure burden. Among the broad spectrum of comorbidities requiring continuous and comprehensive treatment as well as long-term interdisciplinary care, disturbances of cognition, behavior, and communication as well as psychiatric illness are common in children and adults with DRE [4–6]. A variety of brain anomalies, genetic mutations, socio-economic implications, anticonvulsive polytherapy, and inter-ictal epileptiform activity may additionally modify and aggravate the complex course of DRE of various etiologies [3, 4, 6].

In a multi-center trial of 406 patients with epilepsy and primary generalized tonic-clonic seizures and/or focal to bilateral tonic-clonic seizures, 59.6% of patients had experienced at least one seizure-related accidental injury in the last 12 months with the most common being head injuries (35.%) [7]. A quarter of these patients suffered at least one serious injury, and it has been reported that patients with epilepsy are 2.2–4.8 times more likely to die by some type of accident than the standard population [8].

Seizure-associated accidents and injuries further impair quality of life in patients with DRE and may pose an indication for magnetic resonance imaging (MRI). MRI is commonly performed for epilepsy-related injuries or status epilepticus which are major contributors to poor quality of life and mortality. Patients with DRE may also undergo repetitive MRI of the brain (cMRI) for comprehensive pre-surgical evaluation using advanced techniques of MR scanners [9]. Due to the progressive nature of the disease, changes in clinical symptoms may require performing repeated cMRI, direct intracerebral recordings (e.g., stereotactic electroencephalogram [EEG]) [10], functional MRI [11, 12], or novel minimally invasive MRI-guided laser therapies may be needed after VNS implantation [13].

Among adults with depression, more than 25% experience treatment-resistant depression (TRD) that encompasses considerable morbidity and adverse effects on quality of life. TRD comprises failure to respond to two or more antidepressants used at an appropriate dose for an adequate time frame [14]. Since 2001 in the European Union and 2005 in the USA, VNS therapy has also been approved for adjunctive treatment of patients with chronic and recurrent TRD. In addition to standard-of-care treatments with pharmacotherapy, psychotherapy, and electro-convulsive therapy, adjunctive VNS Therapy System has been shown to reduce depressive symptoms, improve quality-of-life, and prevent relapse in patients with TRD [15–17]. Major depressive disorder (MDD) is one of the most diagnosed mental disorders in most first world countries, including Europe, China, and the USA. Structural and functional brain alterations are common in patients with MDD. During the last three decades, MRI has played a critical role in deciphering the pathogenesis of this disorder [18].

Since 1994 in Europe and 1997 in the USA, VNS Therapy System has been an approved and well-accepted adjunctive treatment of patients with drug-resistant epilepsy (DRE). More than 125,000 patients have been implanted with VNS Therapy System (data on file; LivaNova PLC).

VNS Therapy Systems are labeled to allow MRI under certain conditions [19] (Supplementary material); however, no comprehensive real-world experience on the use of MRI in patients with implanted VNS systems is available in the published literature. We conducted this systematic review to analyze information on use and safety of MRI scans in patients with an implanted VNS Therapy System for DRE or TRD.

## Methods

### Search strategy and article selection

A literature review (systematic review) was conducted till June 2020 using the PubMed database (https://pubmed.ncbi.nlm.nih.gov/advanced/). A search syntax strategy was devised using keywords: (“MRI” OR “fMRI” OR “magnetic resonance”) AND (“VNS” OR “Vagus nerve stimulator” OR “Vagal nerve stimulator” OR “Vagus nerve stimulation”). In the query box of the PubMed Advanced Search Builder, the following search query was used: ((MRI[Title/Abstract]) OR (fMRI[Title/Abstract]) OR (magnetic resonance[Title/Abstract])) AND ((VNS[Title/Abstract]) OR (Vagus nerve stimulator [Title/Abstract]) OR (Vagal nerve stimulator [Title/Abstract]) OR (Vagus nerve stimulation [Title/Abstract])). Articles were included if they were manuscripts published in English and described MRI scan procedures on patients with DRE or TRD and implanted with a VNS device for approved indications. Review articles, nonclinical articles, and articles reporting on scans of patients with transcutaneous VNS (t-VNS) were excluded.

This systematic review was conducted in accordance with the Preferred Reporting Items for Systematic Reviews and Meta-Analysis (PRISMA) checklist [20].

### Data extraction

Each article was searched for clear evidence that patients underwent an MRI procedure with a 1.5 T or 3 T MRI scanner with an implanted VNS Therapy System for DRE or TRD and labeled for conditional MRI scanning. The primary endpoint was a positive outcome that was defined as a technically uneventful MRI scan performed in accordance with the VNS device manufacturer guidelines and completed according to the researchers’ planned scanning protocol without harm to the patient.

Each article was searched for any of the following scanning related adverse events:

- Device-related adverse event: Any VNS Therapy System-related non-serious or serious adverse event reported to occur during MRI scanning performed in accordance with manufacturer guideline.
- VNS device malfunction: Reported VNS Therapy System device malfunction during or after MRI scanning performed in accordance with manufacturer guidelines. This could include erratic stimulation or generator malfunction, destruction, or necessary re-programing as confirmed by “system diagnostics” after scanning.

## Results

### Included studies

**Fig. 1:**
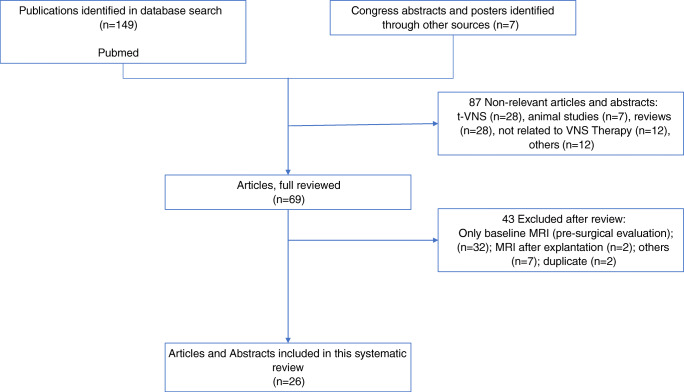
Search flow leading to articles included in this review

The search strategy yielded 156 publications (Fig. 1). Among these publications, 87 were excluded for not reporting on patients with VNS undergoing MRI scans and not being relevant to for the present analysis (e.g., animal studies; t-VNS; patient receives MRI before VNS implantation; not original research (review); not English; “VNS” has other meaning). A full review of the remaining 69 publications led to 43 publications being excluded for reporting only baseline MRI scans in the context of a pre-surgical evaluation, MRI scans after VNS Therapy System explanation, same patients reported in other articles (duplicates), or other reason (e.g., only positron emission tomography [PET] scans performed). Finally, 26 articles were included in this analysis and included data from 216 patients (Table 1). Not all studied specify the field strength of the MRI scanners used, nevertheless 77 patients were reported getting 1.5 T, and 58 patients received 3 T scans. Some studies described in these articles performed multiple scans on the same patient. The duration of the performed scans (exposure time) was only mentioned in a few articles for fMRI sequences and could not be meaningfully evaluated (Table 2).

**Table 1:** Technical specifications of included MRI studies in patients with DRE and TRD receiving adjunctive VNS therapy (details about used scan sequences are listed in Table 2)

Study	Purpose	Design	Field strength	# pts	Age range (yrs)	VNS device	VNS on/off	Coil	Scanned region	Comments
Patients with drug-resistant epilepsy										
Maniker A et al.; Surg Neurol. 2000	Technical safety study	Uncontrolled observational. Prospective	1.5 T	4	N/A	Livanova model 100	On	N/A	N/A	A focus on orientation of implanted generator and “magnet mode”; no MRI-related side effects reported
Narayanan JT et al.; Epilepsia 2002	Evaluation of short-term effects of VNS on brain activation and cerebral blood flow	Uncontrolled observational. Prospective	1.5 T	5	21–57	Livanova model 102	On	Quadrature head coil	Brain	No MRI-related side effects reported
Sucholeiki R et al.; Seizure 2002	Feasibility and safety study of fMRI/BOLD signal intensity from different brain regions during VNS	Uncontrolled observational. Prospective	1.5 T	4	22–49	Livanova model 100	On	T/R head	Brain	No MRI-related side effects reported
Beitinjaneh F et al.; Epilepsia 2002	Elective brain MRI; clinical re-evaluation right mesial temporal sclerosis	Case report	N/A	1	40	Livanova; model N/A	Off	N/A	N/A	First scan interrupted due to seizure; repeated scan completed; no MRI-related side effects reported
Wilfong et al.; Epilepsia 2002	Off-label use; evaluation of gait disturbances	Case report	1.5 T	3	5–14	Livanova model 102	Off	Body	Spine (cervical and thoracic)	No MRI-related side effects reported
Liu WC et al.; J Neurol Neurosurg Psych 2003	Evaluation of activation of brain regions in the left and right hemisphere due to VNS	Uncontrolled observational. Prospective	1.5 T	5	26–40	Livanova model 102	On	N/A	Brain	No MRI-related side effects reported
Tatum WO et al.; Epilepsy Behav. 2004	Emergency use; electroclinical complex partial status epilepticus of right temporal lobe origin	Case report	1.5 T	1	21	Livanova model 102	Off	T/R head	Brain	No MRI-related side effects reported
Roebling R et al.; Epilepsy Res. 2009	Off-label emergency use; rapidly progressive paraparesis	Case report	1.5 T	1	72	Livanova model 101	Off	Body	Spine (cervical)	No MRI-related side effects reported
Gorny KR et al.; J Magn Reson Imaging 2010	Clinical safety study	Uncontrolled observational. Prospective	3 T	17	N/A	Livanova models 100, 102, and 103	Off	T/R head	Brain	No MRI-related side effects reported
Howell KB et al.; Epilepsia 2012	Emergency use; acute and chronic phases of FIRES	Uncontrolled observational. Retrospective	1.5 T or 3 T	1	Child	Livanova; model N/A	N/A	N/A	Brain	Patient died due to FIRES; no MRI-related side effects reported
Stapleton-Kotloski JR et al.; Fr Neurol. 2014	Evaluation of localization of interictal epileptiform activity	Uncontrolled observational. Prospective	N/A	8	6–63	Livanova; model N/A	Off	N/A	Brain	No MRI-related side effects reported
de Jonge JC et al.; Epilepsia 2014	Safety study of epilepsy-related etiology, pre-surgical evaluation; follow-up of tumor pathology; neuronavigation; non-epilepsy-related comorbidities; trauma	Uncontrolled observational. Prospective	1.5 T and 3 T	70	5–68	Livanova; model N/A	Off	T/R head Body	Brain; Extremities; Orbita	4 drop-outs due to abnormal pre-scan device diagnostic (*n* = 2), battery depletion (*n* = 1), and VNS explanation (*n* = 1); suspected post-scan lead break (*n* = 1) probably not related to MRI; body coil accidentally used (*n* = 1): no MRI-related side effects reported; scans with 1.5 T (*n* = 67); scans with 3.0 T (*n* = 30)
Rösch J et al.; Epilepsy Res. 2015	Re-evaluation and follow-up of epileptogenic lesions in mesial temporal lobe	Uncontrolled observational. Prospective	3 T	15	26–72	Livanova model 102	Off	T/R head	Brain	Exclusion of additional cavernomas; Rasmussen encephalitis follow-up; no MRI-related side effects reported
Wang K et al.; Neuropsych Dis Treat. 2016	Evaluation of resting-state brain network after left parietal-occipital lesion-resection surgery	Case report	1.5 T	1	17	PINS Medical model G111	N/A	N/A	Brain	Not a Livanova generator; no MRI-related side effects reported
Jiltsova E et al.; Neuromodulation 2016	Targeting ANT	Uncontrolled observational. Prospective	1.5 T	3	N/A	Livanova; model N/A	N/A	N/A	Brain	No MRI-related side effects reported
Cantarín-Extremera et al.; EU J Paed Neurol. 2016	Late onset of bradycardia and drop attacks	Case report	N/A	1	12	Livanova; model N/A	N/A	N/A	N/A	No MRI-related side effects reported
Lehner KR et al.; J. Neurosurg. 2018	MRI-guided LITT of epilepsy generalized or multifocal seizure onsets	Case report	1.5 T	1	29	Livanova; model N/A	N/A	8-Channel head	Brain	No MRI-related side effects reported
Casimo K et al.; J. Neurosurg Pediatr. 2018	Evaluation of preservation of electrophysiological functional connectivity after partial corpus callosotomy	Case report	1.5 T	1	17	Livanova; model N/A	Off	N/A	Brain	No MRI-related side effects reported
Huang Y et al.; J. Neurosurg Pediatr. 2019	LITT following stereotactic laser ablation for completion corpus callosotomy	Uncontrolled observational. Retrospective	3 T	2	11–40	Livanova; model N/A	N/A	8-Channel head	Brain	No MRI-related side effects reported
Tao et al.; Epilepsia 2020	LITT following stereotactic laser anterior corpus callosotomy (SLACC) for drop attacks in Lennox-Gastaut syndrome	Uncontrolled observational. Retrospective	3 T	9	Median 33	Livanova; model N/A	N/A	N/A	Brain	No MRI-related side effects reported
Zhu J. et al.; Behav Brain Res. 2020	Evaluation of VNS effects on spontaneous brain activity in patients with DRE	Uncontrolled observational. Prospective	3 T	15	Active 19 ± 13; Control 29 ± 3	Livanova; model N/A	Off	T/R head	Brain	No MRI-related side effects reported
Patients with treatment-resistant depression										
Bohning DE et al.; Invest Radiol. 2001	Evaluation of VNS parameter induced BOLD signal changes during synchronized fMRI technique	Uncontrolled observational. Prospective	1.5 T	9	45 ± 8	Livanova models 100 and 101	On	T/R head	Brain	No reports of drop-outs; no MRI-related side effects reported
Lomarev M et al.; J. Psychiatr Res. 2002	Evaluation of VNS parameter induced BOLD signal changes during synchronized fMRI technique	Uncontrolled observational. Prospective	1.5 T	9	50 ± 6	Livanova models 100 and 101	On	T/R head	Brain	Dose-dependent modulating effect of VNS on brain activity; 3 drop-outs due to technical problems: generators did not restart while in the MR scanner; no MRI-related side effects reported; four of the patients participated in a previous study (Bohning et al., 2000).
Mu Q et al.; Biol. Psychiatry 2004	Evaluation of global brain activation due to different VNS parameter	Randomized-controlled. Prospective	1.5 T	12	48 ± 8	Livanova model 102	On	T/R head	Brain	Technical problem with 1 generator: no stimulation signal during scan; 2 patients did not tolerate the scans (not specified); 3 drop-outs
Critchley HD et al.; Psychosom Med. 2007	Evaluation of VNS on emotional memory and its underlying brain activity	Case report	1.5 T	1	48	Livanova; model N/A	On	T/R head	Brain	No MRI-related side effects reported; direct modulatory effects
Nahas Z et al.; Neuropsychopharm. 2007	Evaluation of ventro-medial prefrontal cortex deactivation with greater right insula activation	Randomized-controlled. Prospective	1.5 T	17	Adults	Livanova model 102	On	T/R head	Brain	Panic attack in scanner (*n* = 1); technical problems: due to build-in “magnet switch” from *n* = 107 scans, generator not restarting within scanner (*n* = 11); generator not keeping pre-set duty cycle within scanner (*n* = 16).

Abbreviations: *FIRES*, febrile infection-related epilepsy syndrome; *N/A*, not available

**Table 2:** Addendum to Table 1: technical information about used scan sequences

Author	Sequences
A. Maniker et al.; Surg Neurol. 2000 [1]	fMRI; gradient echo EPI; FOV 24 × 24 cm; TR/TE = 2000/60; 4 slices, 5 mm; matrix 64 × 64; 4 VNS cycles = 20min
D.E. Bohning et al.; Invest Radiol. 2001 [2]	Multi-slice single-shot gradient echo EPI-fMRI; 64 × 64 matrix; FOV = 270 mm; α = 88°; TE = 40.0 ms, slice thickness = 8.0 mm; gap = 0.0 mm; with fat saturation. 15 contiguous 8 mm thickness axial slices, parallel to AC-PC. T1-weighted structural images (TE = 20 ms, TR = 600 ms) for anatomical reference.
J.T. Narayanan et al.; Epilepsia 2002 [3]	Sag T1-weighted (TR 300/TE 14/1 NEX), axial fast spin echo T2 (TR 3000/TE 91/1 NEX), axial fast FLAIR (TR 10002/TE 172/1 NEX) with inversion time (TI) of 2.2 s, axial T1 (TR 500/TE 14/1 NEX), and axial diffusion-weighted echo planar imaging (TR 6000/TE 99-100/1 NEX) with b values of 0 and 1000; 5-mm thickness; gap of 2.5 mm, a 256 × 192 matrix, the same imaging angle along the orbitomeatal line; FOV = 22 or 24 cm. DWI: 128 × 128 matrix size, 5-mm slice thickness with no gap; FOV = 22 × 22 cm; total acquisition time of 42 s. fMRI: EpiBOLD (echo planar blood oxygenation level dependent); single-shot, gradient-echo, echo planar pulse sequence (TR 3000/TE 40), flip angle of 90°, FOV 22 cm, 64 × 64 matrix (slice thickness, 5 mm with no gap). 18 contiguous slices in an axial oblique plane, parallel to the AC-PC line. After fMRI, a routine T1-weighted imaging using the same axial–oblique prescription (TR 500/TE 12/1NEX) was performed to generate corresponding anatomic images for fMRI.
M. Lomarev et al.; J. Psychiatr Res. 2002 [4]	Multi-slice single-shot gradient echo EPI-fMRI; 64 × 64 matrix; FOV = 270 mm; α = 88°; TE = 40.0 ms, slice thickness = 8.0 mm; gap = 0.0 mm; with fat saturation. 15 contiguous 8 mm thickness axial slices, parallel to AC-PC. Also T1-weighted structural images (TE = 20 ms, TR = 600 ms) for anatomical reference.
R. Sucholeiki et al.; Seizure 2002 [5]	High resolution anatomic images, sagittal with the spoiled GRASS pulse sequence with TR = 600 ms, TE = 10 ms, FOV = 24 cm, and matrix size = 256 × 256 fMRI: 12 slices; resting acquisition a time course of images, consisting of 30 s “on” and 30 s “off” for 6 min. Gradient-recalled EPI: TE = 40 ms; FOV = 64 × 64 matrix; 8 mm slice thickness. Other imaging parameters consisted of TR = 2000 ms (flip angle [FA] = 87°); 64 × 64 matrix/8 mm cut thickness yielding voxels of 3.75 × 3.75 × 8 mm.
F. Beitinjaneh et al.; Epilepsia 2002 [6]	n/a
A.A. Wilfong et al.; Epilepsia 2002 [7]	Standard pulse sequences matrix = 256 × 192 and 2 Nex: (1) axial T1-weighted spin echo; TR = 600; TE = 9; (2) coronal T1-weighted fast spoiled GRASS (FSPGR); 60° flip angle; TR = 115, TE = 3.2; and (3) sagittal T1-weighted SE; TR = 400; TE = 8
Liu WC et al.; J Neurol Neurosurg Psych 2003 [8]	fMRI/BOLD; T1-weighted co-planar: TR/TE = 550/min; FOV = 24 cm; matrix = 256 × 256; 28 slices 5 mm slice thickness. fMRI: TR/TE = 4000/60 ms; FOV = 24 cm; matrix = 64 × 64; 28 slices 5 mm slice thickness; 3 scans á 5 min 56 s with 30 s pause between each scan.
Tatum WO et al.; Epilepsy Behav. 2004 [9]	fMRI; DWI; sagT2; axT2; FLAIR; corT2; TR = 10,000 ms and 8000 ms; TE = 107 ms and 83 ms; FOV 40 × 20 cm; 256 × 256 matrix
Mu Q et al.; Biol. Psychiatry 2004 [10]	fMRI/BOLD T1-weighted sagittal; TR = 625 ms; TE = 20 ms; slice thickness = 5 mm; gap = 1 mm; FOV = 256 mm; # slices = 27; matrix = 256 × 256. Whole brain gradient echo planar imaging (EPI): except for a TR = 2279 ms, TE = 45 ms, 64 × 64 matrix, voxel size of 4 × 4 × 6 mm^3^. The fMRI session: 13 min and 40 s.
Critchley HD et al.; Psychosom Med. 2007 [11]	fMRI; normalized T2*-weighted echo planar; T2*-weighted EPI volumes, 2 mm slice thickness, 1 mm interslice gap, bandwidth 2298 Hz/pixel, matrix 64 × 64, FoV 192 mm, TR/TE=3960/50 ms, isotropic spatial resolution 3 mm, 90° flip angle, 30° tilt of the image slice from axial toward coronal orientation to avoid signal dropouts.
Nahas Z et al.; Neuropsychopharm. 2007 [12]	fMRI/BOLD; anatomical T1-weighted sagittal; TR = 625 ms, TE = 20 ms, slice thickness = 5 mm, gap = 1 mm, FOV = 256 mm, number of slices = 27, matrix = 256x256. Whole brain gradient echo planar imaging (EPI): except for a TR = 2837 ms, TE = 45 ms, 128 × 128 matrix, voxel size of 2 × 2 × 6mm^3^. The fMRI session: 400 images, 18 min and 54 s.
Roebling R et al.; Epilepsy Res. 2009 [13]	Sagittal T2-weighted
Gorny KR et al.; J Magn Reson Imaging 2010 [14]	Spoiled gradient echo scans with flip angles of 30° and 60° coronal plane; TR = 6000 ms, TE = 15 ms, 36 cm FOV; 256 × 256 matrix; 5-mm-thick slices. 36 sections in 26 min at each flip angle. 3-plane localizer; sagittal T1-FLAIR, coronal T1 GRE (IR FSPGR or MPRAGE); coronal T2 FLAIR, axial T2 FSE or propeller, axial T2 FSE or propeller, GRE T2*
Howell KB et al.; Epilepsia 2012 [15]	Encephalitis protocol; axial and coronal T2-weighted and FLAIR sequences; T1-weighted volumetric sequence reformatted in three orthogonal planes Epilepsy protocol
Stapleton-Kotloski JR et al.; Fr Neurol. 2014 [16]	n/a
de Jonge JC et al; Epilepsia 2014 [17]	fMRI and others
Rösch J et al.; Epilepsy Res. 2015 [18]	T2 tse cor; T2 ax; IR; DWI ax; T2*ax; MPRAGE; T1 cor; 3D FLAIR
Wang K et al.; Neuropsych Dis Treat. 2016 [20]	rs-fMRI; T1-weighted 3-D magnetization-prepared rapid gradient-echo sequences and functional imaging (echo-planar imaging sequences)
Jiltsova E et al.; Neuromodulation 2016 [21]	MRI examinations with short tau inversion recovery (STIR); T1-weighted magnetization prepared gradient echo (MPRAGE)
Cantarín-Extremera et al.; EU J Paed Neurol. 2016 [22]	n/a
Lehner KR et al.; J. Neurosurg. 2018 [23]	DTI: parallel imaging mode with an acceleration factor of 2. A single shot spin echo planar imaging sequence was used, with 5 images obtained without diffusion weighting and 33 isotropically distributed diffusion gradient directions. The b value in the diffusion-weighted images was 1000 s/mm^2^. The TE was 90.3 ms, and the TR was 14,000 ms, but may have varied up to 14,800 ms in some patients. Images were zero filled to a matrix size of 128 × 128, yielding an image resolution of 0.9 × 0.9 × 3 mm^3^. From the diffusion-weighted images, maps of fractional anisotropy, mean diffusivity, and V1 images (the main vector of the diffusion tensor) were calculated using FSL software. Resting functional MRI: TR 2000 ms, TE 30 ms, matrix 64 ∗ 64, field of view 240 mm, slice thickness 3 mm, and 40 continuous axial oblique slices (1 voxel = 3.75 × 3.75 × 3 mm).
Casimo K et al.; J. Neurosurg Pediatr. 2018 [24]	Anat. MRI and DTI: 20 directions, TR 4.33 s; TE 105 ms, flip angle 90°, slice thickness 4 mm, in-plane resolution 1.56 × 1.56 mm; sagittal T1-weighted MRI: T1-weighted MPRAGE resolution 1.3 × 1.3 mm, 1 mm slice thickness. A 1.5-T MR imager 1.3 mm, 1 mm slice thickness.
Huang Y et al.; J. Neurosurg Pediatr. 2019 [25]	High resolution T1-weighted (3D FSPGR; TE = 3.72 ms; TR = 9.23–9.62 ms, depending on slice coverage; FOV = 240 × 240 mm^2^; acquisition matrix = 256 × 256; voxel size = 0.94 × 0.94 × 1.00 mm^3^; orientation = sagittal), T2-weighted (FLAIR, TE = 88.9 ms, TR = 9500 ms, FOV = 240 × 240 mm^2^, acquisition matrix = 320 × 256, voxel size = 0.94 × 0.94 × 1.00 mm^3^, orientation = axial) and diffusion-weighted images were acquired as part of the standard clinical care for surgical treatment and planning. DT-EPI sequence using ab value of 1000 s/mm^2^ sampling 40 isotropically distributed diffusion directions (dir) (40 dir, b = 1000, b0 = 1, TE = 80.70 ms, TR = 15,000 ms, FOV = 260 × 260 mm^2^, acquisition matrix = 256 × 256, voxel size = 1.02 × 1.02 × 2.50 mm^3^, number of excitations [NEX] = 1). For cases 4, 5, and 6, diffusion data were acquired with a DTEPI sequence (TE = 60.70 ms, TR = 8000 ms, FOV = 250 × 250 mm^2^, acquisition matrix = 256 × 256, voxel size = 0.98 × 0.98 × 2.00 mm^3^, NEX = 1) using a b value of 1000 s/mm^2^ sampling 30 isotropically distributed diffusion directions. For all patients, one additional volume was acquired at b = 0 at the beginning of each scan.
Tao et al.; Epilepsia 2020 [26]	DTI; T1-weighted contrast-enhanced; the number of gradients was 15–32 (median = 32), echo time was 82–136 (median = 96) ms, repetition time was 3.9–9.1 (median = 8.0) seconds, matrix size was 92 × 89 to 128 × 160, slice thickness was 2–5 (median = 2.5) mm, field of view was 224–260 (median = 244) mm, and voxel size was 1.8–2.5 (median = 2.0) mm^3^. A b-value of 1000 s/mm^3^ was used in all cases.
Zhu J. et al.; Behav Brain Res. 2020 [27]	rs-fMRI. A: High-resolution three-dimensional turbo fast spin-echo T1WI sequence (T1W 3D-TFE) with the following parameters: repetition time =12 ms, echo time =5.9 ms, flip angle = 8°, matrix = 256 × 256, field of view = 256 × 256 mm, slice thickness = 1.6 mm, gaps = −0.8 mm, slices = 180, scanning time = 5 min 54 s. B: T2WI 3D FLAIR sequence with the following parameters: repetition time = 5000 ms; echo time = 340 ms; matrix = 252 × 290, field of view = 200 × 232 mm, slice thickness = 1.5 mm, gaps = 1.0 mm, slices = 120, scanning time = 6 min 30 s. C: Single-shot echo planar imaging for the BOLD-fMRI sequence, with the following parameters: echo time = 30 ms, repetition time = 2000 ms, flip angle = 90°, field of view = 224 × 224 mm, image matrix=64 × 64, scanning slice thickness = 3.5 mm, slice gaps = 0.5 mm, slices = 34, scanning time = 8 min.

In 23 of the 26 articles, only cranial MRI (cMRI) was performed. Two studies report on patients receiving spinal MRI and one study includes patients receiving MRI of the extremities. Of the eligible articles, 21 studies evaluated patients with DRE and 5 studies evaluated patients with TRD. Nineteen studies addressed either a technical or clinical research question, two studies addressed MRI in the context of medical emergencies (trauma and febrile infection-related epilepsy syndrome [FIRES]), one study was conducted in the context of a diagnostic workup for bradycardia of unknown origin, and one article reviewed all patients with an implanted VNS receiving MRI at their center for any reason. The remaining 3 studies were published in the last 2 years and addressed MRI scans in the context of laser interstitial thermal therapy (LITT) for DRE.

Of the 26 eligible articles, 25 studies were in patients implanted with VNS Therapy System models from LivaNova PLC, London, UK (before Feb. 2015, Cyberonics, Inc.) and one article presented a case study of a patient implanted with a VNS system from PINS Medical (Beijing, China), which from a design point of view is comparable with the VNS Therapy System. MRI procedures included MRI-guided laser interstitial thermal therapy (LITT) in three patients (Table 1).

### Adverse events

None of the included articles reported a serious adverse event or a device-related adverse event. In one patient with TRD, scanning was interrupted due to a panic attack [11], and this was described as an event of mild intensity.

The article by de Jonge et al. reported one patient, in whom high lead impedance was detected post MRI scanning [21]. However, because “system diagnostics” prior to MR scanning in this patient were not performed according to manufacturer guidelines, no temporal relationship between scanning and lead impedance could be established. In a separate article [22], two patients were reported not to tolerate MRI scanning after previous successful MRI scans; the authors did not specify further details.

Three articles reported one or more generators failing to start stimulation when in the magnetic field of the scanner when MRIs were performed contrary to instructions for use in the physician’s manual [11, 22, 23, 28]. As an example, the design of the study by Nahas and colleagues was based upon achieving uninterrupted VNS at programmed device settings during functional magnetic resonance imaging (fMRI). To achieve this, Model 101 VNS Therapy Systems were programmed to continue during MRI. Instructions for use call for programming current output to 0 mA before MRI (Physician’s Manual, 2002). During the study, all Model 101 VNS Therapy Systems performed as designed. This included resetting of stimulation parameters to factory programmed settings when exposed to magnetic and radiofrequency (RF) fields generated during MRI, or movement of the pulse generator’s reed switch when exposed to a static magnetic field to interrupt the programmed Model 101 duty cycle. While this caused inability for the investigators to maintain uninterrupted VNS during fMRI and resulted in a number of scans having to be excluded from the final analysis, no stimulation-related adverse events during fMRI and no unintentional resetting of VNS parameters to a higher output by fMRI were reported [11].

There were no reports of adverse events in children with VNS Therapy System during MRI examinations. In three case reports, the unintentional execution of a full-body MRI in patients implanted with an VNS Therapy System was described, with no reported clinical consequences for the patients [21, 24, 25].

Taken together in this analysis, one non-serious adverse event was reported among all reported patients (0.4%) and was unrelated to the VNS system implanted. No serious adverse events were reported. No VNS Therapy System malfunction was reported when MRI scanning was performed according to instructions for VNS Therapy System use in the physician’s manual.

## Discussion

Clinical concerns during MRI scanning of patients with an implanted VNS Therapy Systems are typically focused based on three phenomena during MRI: heating, force, and malfunction. These could be caused by the interaction of the VNS Therapy System with the static fields, gradient fields, and the RF fields generated by the MRI scanner. The static field can exert torque and force on ferromagnetic objects, which could result in displacements; RF and gradient fields could each result in excessive heating, and all three of these fields could either separately or in combination theoretically cause generator vibration and malfunction. Clinically, these would be manifested by the presence of pain or loss of therapy. In addition to these hazards, there is also the possibility of unintended stimulation caused by either the RF or gradient fields.

Laboratory testing focusing on the functional aspects of the VNS Therapy System indicated that MRI procedures performed at 1.5 T and 3 T produced no significant alterations in the operation of the generators (Livanova, data on file). These findings coincide with results described by Shellock and coworkers for the VNS Therapy System studied under in vitro conditions in 1.5 T and 3 T scans [19]. However, Shellock and coworkers identified potentially unsafe conditions with regards to MRI-related heating, therefore MRI scans at 1.5 T and 3 T in patients implanted with VNS Therapy System should only be performed respecting the clearly defined “exclusion zones” in the manufacturers’ guidelines [19, 28].

None of the here reviewed literature on MR imaging studies in patients implanted with the VNS Therapy System did not report any symptoms or signs of pain, local discomfort, or loss of therapy. However, most are brain studies and have scanned fMRI, DTI, and 3D T1 (MP-RAGE or FSPGR), of which all are low SAR sequences. Two studies involving MRI of the spine reporting 4 patient scans in total. Especially, the work of Wilfong [24] where 3 patients underwent MR spine imaging must be assessed critically, as this is an abstract presumably concerns a conference contribution and is not widely available.

Our analysis here has demonstrated that cranial magnetic resonance imaging for soft tissue visualization can be performed safely under appropriate conditions in patients implanted with a VNS Therapy System. Such “MRI conditional” use of MRI means that a VNS Therapy System poses no known hazard in a specified magnetic resonance environment if specific conditions that are described in the physician’s manual are met (Supplemental materials).

MRI scans of the head, neck, and extremities are possible currently with 1.5 T and 3 T MRI scanners. Initial studies have demonstrated that 7 Tesla MRI may improve lesion detection in epilepsy patients [26, 27, 29, 30]. Comprehensive technical assessment will be needed in order to evaluate the safety of MRI scanning at this higher magnetic field strength in patients implanted with a VNS Therapy System.

VNS has been established as an effective, safe, and well-tolerated adjunctive therapeutic option in patients with DRE [31] and TRD [32]. This present systematic review on real-world use of MRI in patients with DRE and TRD has revealed no serious adverse events, device-related adverse events, or VNS Therapy System malfunction when MRI scanning is performed in accordance with VNS Therapy System instructions for use in the physician’s manual (LivaNova PLC, 2019; Supplemental materials).

With the first approval for VNS therapy (1994 EU and 1997 USA, Cyberonics Inc.), MRI scans were only allowed using local transmit/receive (T/R) coils. T/R coils are commonly used only for extremity imaging on modern MRI scanners. Brain MRI scan protocols nowadays often use parallel imaging and cannot be scanned unmodified using a T/R head coil. Since 2017, the MRI guidelines for VNS therapy were expanded and permit the usage of a transmit body coil, together with a receive-only local coil, respecting the International Electrotechnical Commission (IEC 60601-2-33) SAR limits (2 and 3.2 W/kg for whole body and head scans, respectively) under normal mode operation (Group A, Supplemental materials) [28]. According to these guideline extensions, 28 out of 216 patients may have been scanned with transmit body coil.

### Limitations

This systematic review must be interpreted with caution as it is retrospective and evaluated a heterogenous mix of patient populations, VNS systems, and MRI techniques and methods. Most of the articles summarized experience that was based upon a small number of patients and had a length of long-term follow-up that differed across studies. These limitations notwithstanding, no safety signal emerged from this systematic literature review of real-world MRI use in patients implanted with a VNS Therapy System when MRI is performed according to instructions for MRI system use (LivaNova PLC, 2019).

### Conclusion

This systematic review indicates that cranial MRI of patients with an implanted VNS Therapy System can be completed satisfactorily, is tolerable, and safe using 1.5 T and 3 T scanners, when the manufacturer guidelines are followed for MRI scanning.

## Supplementary Information

ESM 1 (PPTX 174 kb)

ESM 2 (PPTX 76 kb)

## Abbreviations

cMRI: Cranial MRI
DRE: Drug-resistant epilepsy
fMRI: Functional magnetic resonance imaging
LITT: Laser interstitial thermal therapy
MDD: Major depressive disorder
MRI: Magnetic resonance imaging
PRISMA guidelines: Preferred Reporting Items for Systematic Reviews and Meta-Analyses
TRD: Treatment-resistant depression
VNS: Vagus nerve stimulation
DTD: Difficult-to-treat depression
RF: Radio frequency
