# Supplementary material for: A systematic review of magnetic resonance imaging in patients with an implanted vagus nerve stimulation system
Source: Neuroradiology. 2021 Apr 12;63(9):1407–17. doi: 10.1007/s00234-021-02705-y (PMC8376717; doi:10.1007/s00234-021-02705-y)
Supplement: Supplementary file 1 — (PPTX 174 kb) [file 234_2021_2705_MOESM1_ESM.pptx]

## Slide 1
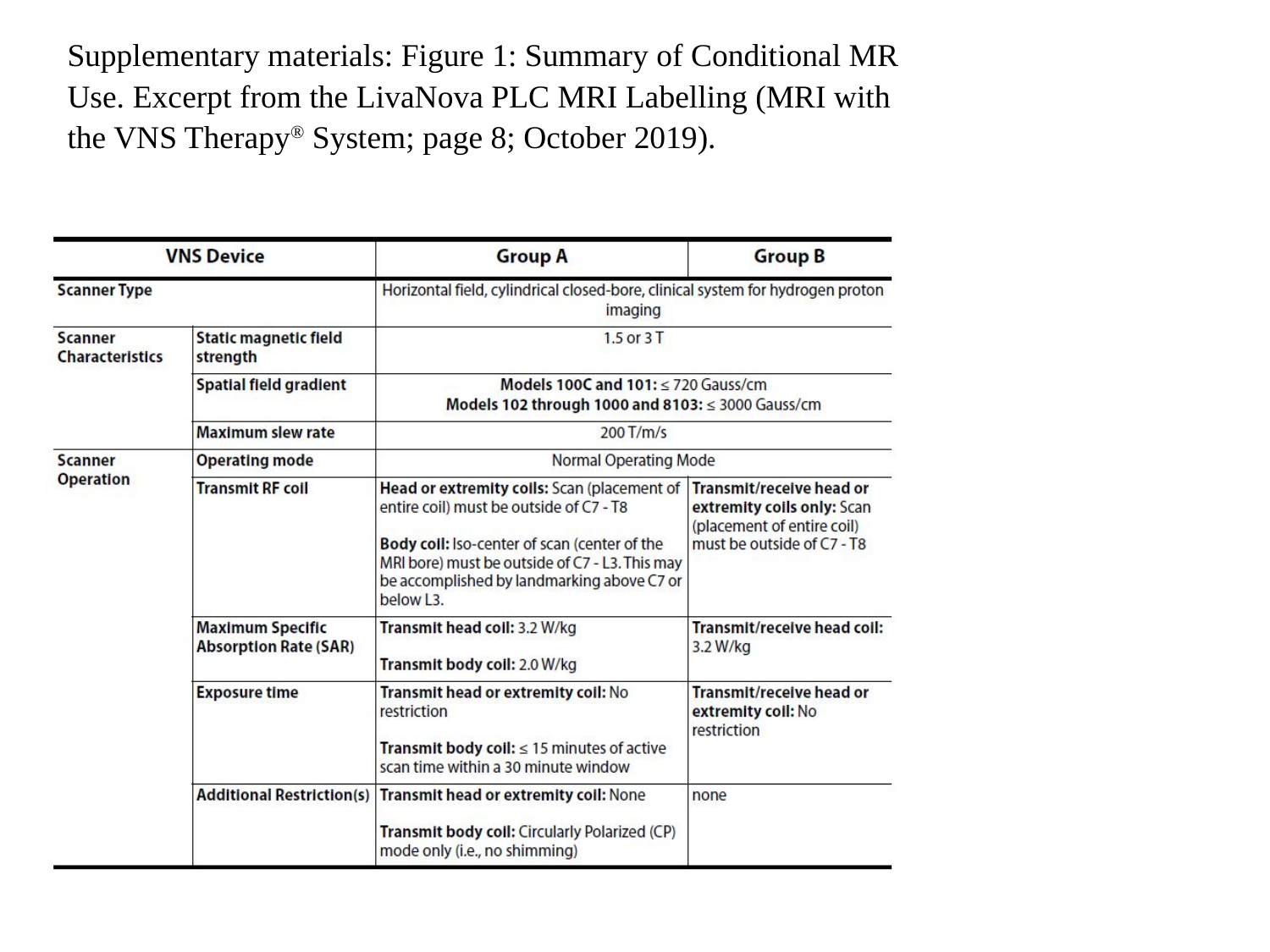

Supplementary materials: Figure 1: Summary of Conditional MR Use. Excerpt from the LivaNova PLC MRI Labelling (MRI with the VNS Therapy® System; page 8; October 2019).
