# Supplementary material for: A systematic review of magnetic resonance imaging in patients with an implanted vagus nerve stimulation system
Source: Neuroradiology. 2021 Apr 12;63(9):1407–17. doi: 10.1007/s00234-021-02705-y (PMC8376717; doi:10.1007/s00234-021-02705-y)
Supplement: Supplementary file 2 — (PPTX 76 kb) [file 234_2021_2705_MOESM2_ESM.pptx]

## Slide 1
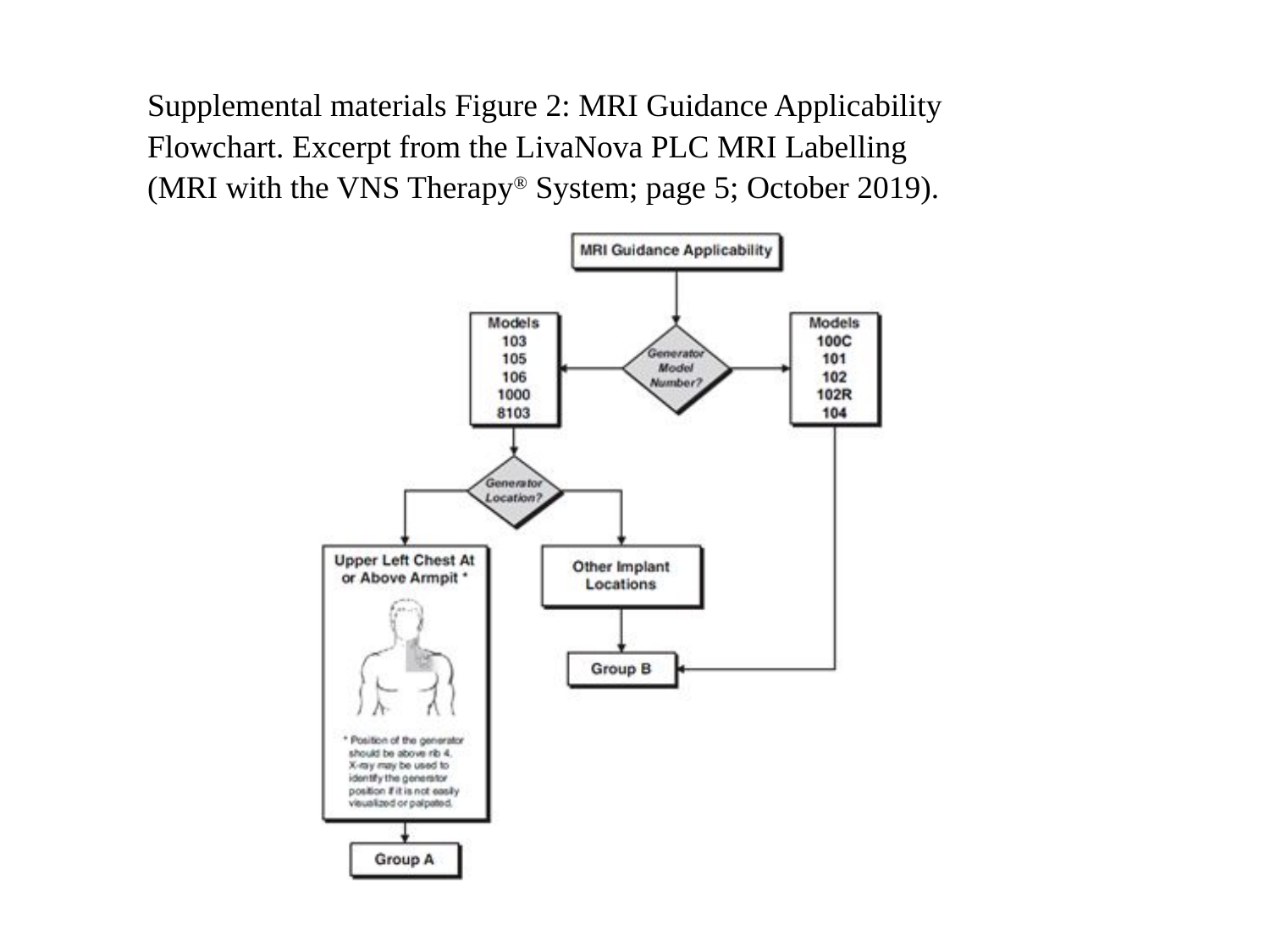

Supplemental materials Figure 2: MRI Guidance Applicability Flowchart. Excerpt from the LivaNova PLC MRI Labelling (MRI with the VNS Therapy® System; page 5; October 2019).
